# The Future of DNA Adductomic Analysis

**DOI:** 10.3390/ijms18091870

**Published:** 2017-08-29

**Authors:** Peter W. Villalta, Silvia Balbo

**Affiliations:** 1 Masonic Cancer Center, University of Minnesota, Minneapolis, MN 55455, USA; balbo006@umn.edu; 2 Division of Environmental Health Sciences, University of Minnesota, Minneapolis, MN 55455, USA

**Keywords:** DNA adducts, DNA adductomics, DNA damage, genotoxicity, chemical carcinogenesis, high resolution accurate mass (HRAM) mass spectrometry, constant neutral loss

## Abstract

Covalent modification of DNA, resulting in the formation of DNA adducts, plays a central role in chemical carcinogenesis. Investigating these modifications is of fundamental importance in assessing the mutagenicity potential of specific exposures and understanding their mechanisms of action. Methods for assessing the covalent modification of DNA, which is one of the initiating steps for mutagenesis, include immunohistochemistry, ^32^P-postlabeling, and mass spectrometry-based techniques. However, a tool to comprehensively characterize the covalent modification of DNA, screening for all DNA adducts and gaining information on their chemical structures, was lacking until the recent development of “DNA adductomics”. Advances in the field of mass spectrometry have allowed for the development of this methodology. In this perspective, we discuss the current state of the field, highlight the latest developments, and consider the path forward for DNA adductomics to become a standard method to investigate covalent modification of DNA. We specifically advocate for the need to take full advantage of this new era of mass spectrometry to acquire the highest quality and most reliable data possible, as we believe this is the only way for DNA adductomics to gain its place next to the other “-omics” methodologies as a powerful bioanalytical tool.

## 1. Introduction

Covalent modification of DNA plays a key role in the initiation phase of chemically induced carcinogenesis [1,2]. Modifications, typically referred to as DNA adducts, if not repaired can lead to genomic instability that may result in mutations, which can translate into altered gene expression, abnormal cell growth, and disruption of normal cell function [1,3,4]. Therefore, the measurement of DNA adducts is of fundamental importance in assessing the potential carcinogenic effects of specific exposures and understanding their mechanisms of action. Additionally, the characterization of this type of DNA damage is extremely valuable for the investigation of the safety of exposure to substances used in the industrial and manufacturing processes, pharmaceuticals, environmental pollutants, as well as life-style factors associated with increased cancer risk.

The identification and structural elucidation of DNA adducts in human tissues can be used to either identify specific exposures which resulted in genotoxicity or confirm that suspected exposures have occurred and led to DNA modification. Additionally, the identification and/or quantitation of DNA adducts can reveal important mechanistic aspects of cancer etiology, by elucidating the sequence of events occurring from human chemical exposure to DNA modification, and ultimately to the occurrence of a tumor [3,5]. Therefore, methods to detect and recognize these specific alterations can provide insight into the type of DNA damage resulting from the exposure studied and can provide opportunities for the design of more efficient intervention and prevention approaches.

There are several established methods for assessing the genotoxicity and mutagenicity induced by exposure to various compounds. The in vitro metaphase chromosome aberration assay, the in vitro micronucleus assay, and the mouse lymphoma gene mutation assay (MLA) are widely used and can be considered sufficiently validated. These three assays are currently considered equally appropriate for measurement of chromosomal damage when used together with other genotoxicity tests in a standard battery for testing for example pharmaceuticals. In vivo tests are included to account for absorption, distribution, metabolism, and excretion, with the analysis either of micronuclei in erythrocytes, or of chromosome aberrations in metaphase cells in bone marrow, currently being the most frequently used [6]. In vitro and in vivo tests that measure chromosomal aberrations in metaphase cells can detect a wide spectrum of changes in chromosomal integrity. These methods give a general overview of the DNA damage resulting from an exposure, however they do not provide specific information on the chemical structure of the modifications the damage may result from, or on the mechanism through which the damage may have occurred. DNA adducts analysis has the ability to provide this critical information.

Methods to directly detect and quantify DNA adducts in humans have been developed in the past 30 years, with immunohistochemistry, ^32^P-postlabeling, and mass spectrometry-based techniques being the most common [7,8]. Among these, only ^32^P-postlabeling has been used for DNA adduct screening with varying degrees of comprehensiveness, but lacking the ability to provide information on the specific chemical nature of the DNA adducts detected. Immunohistochemistry methods [3,9] rely on specific antibodies for detection of a particular adduct or type of adduct. Examples include polycyclic aromatic hydrocarbon-DNA adducts assayed using the monoclonal 5D11 antibody [10] and cisplatin-DNA adducts using rabbit antiserum NKI-A59 against cisplatin-modified calf thymus DNA [11]. Liquid chromatography-tandem mass spectrometry (LC-MS^2^) has become the preferred technique for targeted DNA adduct analysis [12,13,14,15,16]. The popularity of this approach is due to its highly selective nature, sensitivity rivaling and at times surpassing that of ^32^P-postlabeling, and the ability to perform accurate quantitation using stable isotope dilution [12,13,14,15,16]. Both endogenous adducts, including those related to epigenetic modifications, and exogenous adducts resulting from nucleobase alkylation, oxidation, deamination, and cross-linking due to various exposures, have been measured (see Section 3.2, Figure 3 for representative examples) [15,16]. However, traditionally LC-MS^2^ approaches have focused on the analysis of a limited number of DNA adducts at a time, which does not allow them to provide a global picture of the DNA modifications resulting from an exposure or a combination of exposures. The ideal assessment of the potential DNA modification induced by the combination of various exposures requires a methodology which is capable of screening for adducts in a global and comprehensive fashion with as much structural information as possible.

## 2. Conventional Approach for DNA Adduct Screening: ^32^P-Postlabeling

The ^32^P-postlabeling methodology is well-suited to broad-based DNA adduct screening because of its ability to monitor many adducted nucleotides simultaneously in a given sample [17] and its high sensitivity with certain DNA adducts detectable at levels approaching 1 adduct per 10^10^ nucleotides. Adducts are identified either as spots on thin layer chromatography plates observed by autoradiographic detection or as peaks using high-performance liquid chromatography (HPLC) separation with radioactive detection. This method has been successfully employed to screen for DNA adducts in a variety of human tissues and white blood cells [18,19,20,21,22,23], in exfoliated epithelial cells in urine of smokers [24], in breast milk of lactating mothers [25], and the sputum of lung cancer patients [26,27]. These studies have revealed that human DNA is modified by many different electrophiles, including those formed endogenously as well as by both environmental and dietary genotoxicants. It has also been shown that the level of DNA modification can be influenced by lifestyle and host factors [20,28]. The ^32^P-postlabeling methodology, however, does have some significant limitations [29], including being labor-intensive, needing significant amounts of radioactive phosphorus, and having potentially highly variable labeling efficiency [30]. Additionally, the most significant limitations are the lack of information regarding the structure of the DNA adduct detected and, at times, the presence of co-migrating adducts on the thin layer chromatography plate [19,31], both of which make the chemical structural determination of adducts very difficult.

## 3. New Approach for DNA Adduct Screening: DNA Adductomics Using Liquid Chromatography-Mass Spectrometry (LC-MS)

Ideally, what is required to comprehensively assess covalent modification of DNA in realistic scenarios, with various exposures, associated metabolism, and downstream endogenous effects, is an approach which combines the screening capability of ^32^P-postlabeling and the structural information provided by targeted LC-MS^2^ analysis, with little or no a priori assumptions regarding the nature of the adducts formed. An effort to address this need has led to the establishment of the field of LC-MS^n^-based DNA adductomics intended to comprehensively screen for DNA modifications, including both known DNA adducts and those which have not been previously detected and/or identified. The first example of this basic approach that we are aware of was performed by Claereboudt and coworkers in 1990 [32], but its further development in subsequent years was limited by the sensitivity and selectivity of the available instrumentation. The rapidly improving instrumentation and technology of the past 10 years has paved the way for the development of more robust DNA adductomics approaches, able to perform a comprehensive characterization of the chemical nature of DNA modification. The field of DNA adductomics [29], while still in its infancy, has now become significantly more powerful with new approaches [33,34,35,36,37,38] taking advantage of modern mass spectrometry and the wide spread use of high resolution mass spectrometers, allowing for the elucidation of the chemical formula of adducts and their fragments. Details on the advantages of using high resolution mass spectrometry are described further in Section 3.4.2. As with any “-omics” based screening technique, DNA adductomics presents new analytical challenges and therefore requires development work aimed at optimizing chromatography, sample preparation, and data collection and analysis. The approaches this field is pursuing and the features and challenges they each present are described here, with special emphasis on the need for the development of robust, reliable and effective DNA adductomic methods.

### 3.1. Typical DNA Adductomics Workflow

**Figure 1:**
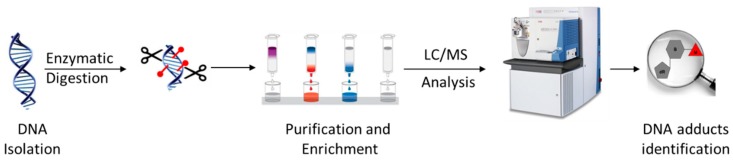
Summary of a typical sample preparation and analysis workflow for DNA adductomics analysis.

The sample preparation for the basic LC-MS^n^-based DNA adductomics workflow (Figure 1) is similar to that typically performed for targeted LC-MS^2^ DNA adduct quantitation, with some modifications to make it more general so as to avoid the potential loss of unknown adducts during sample preparation [15,16]. First, DNA is isolated from the sample, typically tissue, cells, or blood, and usually hydrolyzed to nucleosides using a cocktail of enzymes or to nucleobases by mild acid treatment. When enzymes are used, they are often removed through protein precipitation using organic solvent or through the use of a molecular weight filter cartridge. Salts and other hydrophilic substances are commonly removed by solid phase extraction or fraction collection off of an HPLC column. The resulting samples are typically concentrated through drying and reconstitution to a small volume. The resulting samples are usually analyzed with LC-MS^2^, utilizing a key feature of the fragmentation behavior of modified nucleosides, which is discussed in some detail below.

### 3.2. Key Feature of the Positive Ion LC-MS^n^ DNA Adductomics Methodology

**Figure 2:**
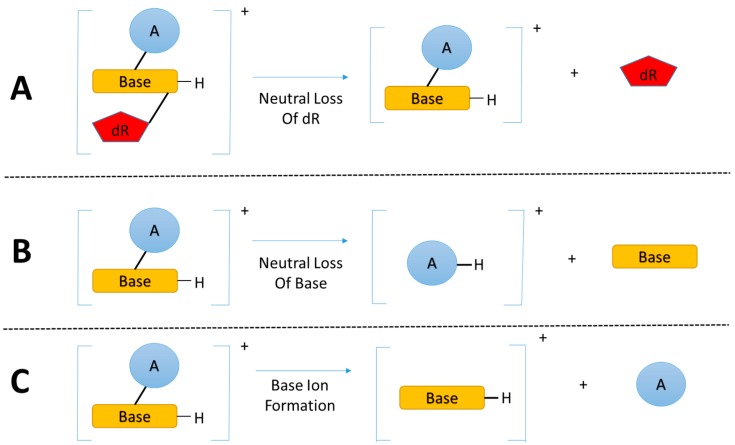
(**A**) The dominant fragmentation pathway of nucleoside adducts is the neutral loss of the 2’-deoxyribose moiety; (**B**) and (**C**) Common fragmentation pathways of nucleobase adducts. (Base = nucleobase, A = modification, and dR = 2’-deoxyribose).

The enzymatic hydrolysis of modified DNA results in the liberation of modified nucleoside adducts (DNA adducts) which share the same basic chemical structure, the modified nucleobase linked to a deoxyribose (dR) group. The primary and critical feature for DNA adductomic screening of nucleoside adducts is the nearly universal neutral loss (*m*/*z* 116 amu) of the dR moiety, upon fragmentation (MS/MS) of the positive ion of the precursor, as shown in Figure 2A [29]. This feature allows for the identification of a given trace level adduct from the multitude of more abundant chemical noise ions present in the LC-MS chromatogram of a given sample.

A recent study [37] expanded the DNA adductomic approach by combining the neutral loss of the bases (Figure 2B), a common ion fragmentation pathway of base adducts, with the conventional neutral loss of dR, allowing for the simultaneous screening of nucleoside adducts and aglycone base adducts. Aglycone base adducts can result upon loss of the deoxyribose from unstable nucleoside adducts upon enzymatic or thermal hydrolysis of the DNA, i.e., N7 position of guanine, N7/N3 of adenine, and the O^2^ positions for both cytosine and thymine [39]. This ion fragmentation pathway (Figure 2B) can be very useful to broaden the basic DNA adductomic approach [37].

Another DNA adductomic analysis [40], which allows for the detection of guanine adducts, takes advantage of the fact that aglycone guanine adducts often fragment to form *m*/*z* 152 (guanine + H^+^) and 135 (guanine-NH_3_ + H^+^) ions. This observation suggest that a similar detection scheme could be used for adenine adducts with characteristic fragments of *m*/*z* 136 (adenine + H^+^) and 119 (adenine-NH_3_ + H^+^). It seems likely that this approach could be broadened to include all four bases (Figure 2C) and would be complimentary to the neutral loss of bases (Figure 2B), allowing for the detection of the majority of aglycone base adducts.

The three fragmentation pathways outlined in Figure 2 could in theory be combined for a nearly comprehensive DNA adductomics methodology for enzymatic hydrolyzed DNA, allowing for the detection of both nucleoside DNA adducts as well as any base DNA adducts resulting from the loss of the deoxyribose group from unstable nucleoside adducts. It would be possible to combine all three fragmentation pathways into a data dependent MS^3^ or MS^2^ approach or a data independent MS^2^ approach. These scanning modes are discussed below. Also, ion fragmentation pathways of the aglycone base adducts (Figure 2B,C) could be used for a DNA adductomic method screening for adducts formed upon DNA acid hydrolysis where the deoxyribose group is cleaved and only the modified base is present. A better understanding of the fragmentation of aglycone base adducts would be very useful in understanding the comprehensiveness of this approach and in confirming the identity of the features resulting from the analysis. The most widely used MS-based “omics” methodologies, proteomics and metabolomics, rely on vast databases for data analysis, which allows for an organized and automated workflow for data analysis and interpretation. DNA adductomics is lacking similar automated data analysis and bioinformatic tools and, therefore, we envision that efforts devoted to create a database containing fragmentation spectra of aglycone base and nucleoside adducts will be extremely helpful in advancing the field of DNA adductomics.

**Figure 3:**
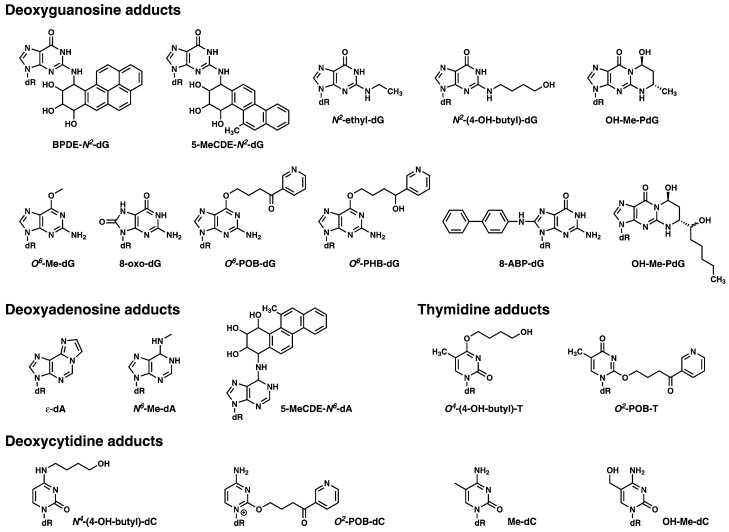
Examples of the type of DNA modifications that can be analyzed with mass spectrometry-based DNA adductomic approaches. The deoxyribose moiety of the structure is abbreviated as dR.

A list of DNA adducts which are representative of those which could be screened for using DNA adductomics, and which have been extensively studied using mass spectrometry based approaches in a variety of experimental settings, are shown in Figure 3.

### 3.3. A Sensitive and Selective LC-MS^n^ Screening

**Figure 4:**
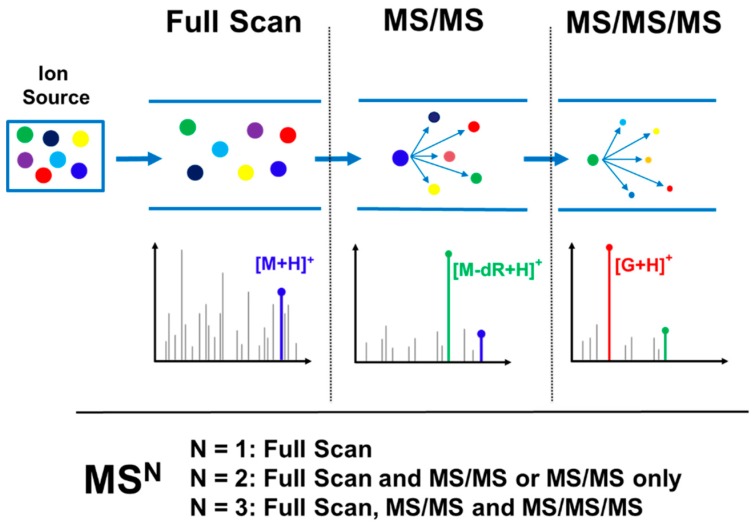
Illustration of different types of high resolution accurate mass (HRAM) DNA adductomic (MS^n^) detection where N = 1 represents Full Scan, N = 2 represents Full Scan and MS/MS or MS/MS only, and N = 3 represents Full Scan, MS/MS, and MS/MS/MS. In this example, M is a nucleoside adduct with the general formula of M = X-G-dR where G is guanine, dR is the deoxyribose moiety, and X is the modification. In the Full Scan panel, the accurate mass of the DNA adduct ([M + H]^+^) can be extracted to generate a chromatogram and provide molecular formula information (this step is common to both DDA and DIA approaches). In the second panel, the MS/MS signal can either be extracted to generate a chromatogram (as in the case of a DIA approach) or provide MS/MS spectral data for the adduct (as in the case of a DDA approach). Finally, in the third panel, the MS/MS/MS fragmentation data can be used to indicate presence of a DNA adduct as well as provide structural confirmation/information (this is the final step for the DDA approach, while it is done in a separate injection in a DIA approach, focusing on candidate adducts identified in the first analysis).

Probing for DNA adduct formation in human samples requires maximum sensitivity due to the trace levels of these analytes (1 adduct in 10^6^–10^10^ nucleotides) in often limited amounts of available DNA (typically 1–100 μg DNA); likewise, in cells or animal models, sensitivity is critical due to the need to keep dose levels low to approximate human exposure. High selectivity in adduct identification is also needed to differentiate DNA adducts from the significant background signal present in biological samples. The need for optimal sensitivity and selectivity is even greater than what is required for trace level targeted DNA adduct quantitation [15] due to the need to screen for multiple adducts, often of unknown identity, across large mass ranges and lacking isotopically labeled internal standards and the well characterized fragmentation patterns of the targeted DNA adduct analytes. This requirement for greater sensitivity and selectivity means that successful analysis is only possible when taking advantage of the technological advancements and scanning modes available with the latest generation of instrumentation. This is particularly true for the use of high resolution accurate mass (HRAM) MS^n^ detection, which is the acquisition of spectral data with typical mass resolving power sufficient to differentiate masses within 0.01–0.001 amu of each other and accuracy of mass measurement on the order of 0.001 amu, often sufficient to determine the molecular formula of the ion. This type of data acquisition greatly increases both the specificity of the analysis, allowing for precise characterization of the detected adducts, as well as increased sensitivity due to the ability to differentiate the adduct ion signals from isobaric background ions signals. The acquisition of HRAM DNA adductomics data can be performed by MS^1^ mode consisting of full scan data, or MS^2^ mode consisting of full scan and MS/MS mass spectral data, or MS^3^ mode consisting of full scan, MS/MS and an additional fragmentation level (MS/MS/MS). Figure 4 illustrates this variety of data acquisition modes in the context of adduct screening.

### 3.4. Rapidly Evolving Technology

The technological capabilities of mass spectrometers, propelled by the development of electrospray and MALDI (matrix-assisted laser desorption/ionization), have been improving rapidly for more than two decades. Further driving the improvements, over the past 15 years or so, has been the development and promise of proteomics. These two factors have resulted in the development of powerful, targeted small molecule and macromolecule quantitative and qualitative analytical capabilities as well as new -omic analyses, including metabolomics and lipidomics. DNA adductomics has now joined the list of -omic methodologies which are used to investigate biological systems. The improvement in technology is continuing unabated with steady advances occurring yearly (e.g., improved ion trap, quadrupole-trap, and quadrupole-TOF instrumentation) with the occasional quantum leap forwards such as the introduction of Orbitrap technology (Thermo Scientific), rapid scanning Q-TOF technology (AB Sciex Triple-TOF), powerful ion mobility capabilities (Waters Synapt technology), and advanced hybrid instruments (Thermo Scientific Fusion instrumentation).

#### 3.4.1. Nanospray Ionization

Electrospray ionization and sampling efficiency increases dramatically as the flow rate is decreased. Proteomics takes advantage of this phenomenon by operating in “nanoflow” ionization mode with flow rates in the 100 s of nanoliters per minute. Nanospray operation has evolved from exotic—requiring flow splitting, handmade emitters, and self-packed columns, and the need to master using delicate low flow fittings and tubing—to truly routine, with the use of commercially produced ultra performance liquid chromatographs (UPLCs) designed for nanoflow operation, easy to use nanospray sources, and pre-made nanoflow columns. It is now possible with minimal training for new analysts to easily work in this mode, and has been used in the field of LC-MS^n^ DNA adduct analysis, including DNA adductomics [36,37,38,41]. Due to the trace levels of DNA adducts and the need to screen for multiple and often unknown adducts, maximizing sensitivity is critical to successful DNA adductomic analysis.

#### 3.4.2. High Resolution Accurate Mass (HRAM) Data

The ability to measure the mass of adducts with accuracies [16] sufficient to provide the selectivity necessary to discriminate them from chemical noise, as well as provide significant information regarding identity of the adducts, has become increasingly possible with recent advances in Orbitrap and Q-TOF instrumentation. The power of HRAM data acquisition comes from the combination of high resolution, which allows ions of similar mass to be resolved from each other, and the subsequent accurate mass measurement allowing for the precise measurement of the ion masses. Without sufficient resolution, only ions which dominate in intensity over adjacent unresolved ions can be measured accurately. With trace level analysis, the analyte ions of interest often have much lower intensity relative to background ions with similar *m*/*z* values, and this explains the need for higher levels of resolution. This is especially true in the case of MS^1^ data where the number of background ions is dramatically larger than present in MS^n^ fragmentation spectra, where the initial parent ion isolation dramatically reduces the number of ions which need to be resolved in the acquired spectra. When sufficient mass resolution is used for the selective detection of trace level DNA adducts, the accurate mass measurements can often provide sufficient information to determine the molecular formula of the analyte and fragment ions, especially when using internal lock masses for maximum accuracy and accounting for the abundances of their isotopic peaks.

#### 3.4.3. Scanning Modes for HRAM MS^n^ Data Acquisition

New instrumentation has made new operational modes possible. Early DNA adductomics [29] primarily utilized triple quadrupole instrumentation to perform neutral loss and pseudo-neutral loss screening, whereas more recent analyses have taken advantage of HRAM instrumentation for analyses. For example, Orbitrap detection with data dependent acquisition (DDA) of HRAM MS^1^, MS/MS, and MS/MS/MS data has been performed [36,37,38]. DDA analysis has been a mainstay of LC-MS^2^ proteomic analysis, however recently a new scanning mode, data independent acquisition (DIA), has become popular and made possible initially by faster scanning Q-TOF instruments, and more recently by faster scanning Orbitrap detectors. This scanning mode has recently [35] been utilized for DNA adductomics and will be discussed below along with a brief description of DDA, and their merits with regard to DNA adductomics will be addressed.

#### 3.4.4. Data Dependent Acquisition (MS^n^)

Data dependent acquisition (DDA) is a continuous scanning mode in which each full scan spectrum acquired is followed with multiple subsequent MS/MS fragmentation events with rapid, on-the-fly precursor ion selection by the instrument software. The detection, and possibly identification, of adducts is done analyzing the product ion spectra using the DNA adduct fragmentation types discussed above. This data acquisition mode was developed for shotgun proteomic analysis and is the conventional approach for this type of analysis. There are many features which are available for tailoring this scanning mode to the specific analysis, including those typically used for proteomics such as dynamic exclusion, exclusion lists, charge state selection, monoisotopic precursor selection, etc. as well as others more likely to be used for small molecule analysis such as inclusion lists, neutral loss triggering, product ion triggering, etc. The current sophistication of most LC-MS instrumentation makes programming of methods using this scanning mode straightforward, although there are typically many parameters which need to be optimized for a particular sample type and experiment. In contrast to proteomics, there are no software tools available for the automated analysis of the resulting data and it must be analyzed manually.

#### 3.4.5. Data Independent Acquisition (MS^2^)

Data independent acquisition (DIA) in its simplest terms is the acquisition of fragmentation spectra for all ions across a broad mass range rapidly enough to acquire multiple data points across the chromatographic peak shape of the analytes of interest. There are various forms of instrument scanning modes for acquisition of DIA data, typically with concurrent acquisition of the corresponding full scan data [42]. This approach [43,44,45] was developed as an alternative to the conventional proteomics approach of DDA, and has gained popularity; more recently researchers have started to implement it in the acquisition of metabolomics data [46,47]. The aim of DIA is to comprehensively fragment all analytes of interest present, thereby providing for a complete data set, in contrast to DDA, which uses ion intensity as a criterion for fragmentation and is prone to missing the detection of lower level analytes. This makes DIA amenable to comprehensive detection/quantification of adducts, either in a targeted fashion by extraction of parent and product ions from the full scan and MS/MS data, respectively, or in an untargeted fashion by relying on peak picking software to identify chromatographic peaks with the correct fragmentation characteristics. Both the targeted and untargeted analysis require co-elution of full scan and MS/MS chromatographic peaks as a criteria for adduct detection. Software and bioinformatics tools are required to handle the challenging amount of data produced if the promise of DIA is to be fulfilled. The DIA approach is rapidly evolving with significant progress being made, primarily in the field of proteomics [42,44,48], but adapting the approach for DNA adductomics will take significant development work to take advantage of the possibilities of the approach.

#### 3.4.6. DDA and DIA for DNA Adductomics

The DDA and DIA scanning modes, which we feel take full advantage of the available instrumentation for DNA adductomics, are the constant neutral loss with triggering of MS/MS/MS fragmentation (CNL/MS^3^) mode and wide range selected ion monitoring with corresponding MS/MS fragmentation (Wide SIM/MS^2^) mode, respectively. The features characterizing these modes of operation are summarized in Table 1. Briefly, the DDA-CNL/MS^3^ approach utilizes the triggering of MS/MS/MS fragmentation upon observation of the neutral loss of the mass used to identify adducts (typically deoxyribose, *m*/*z* 116.0473). This analysis provides the advantage of relatively straightforward data analysis, although software tools providing automated analysis are still lacking, as well as a rich set of fragmentation data (both MS/MS and MS/MS/MS) for adduct verification and/or identification in a single injection. The primary negative with this approach is the potential for incomplete sampling due to insufficient speed of analysis, resulting in low level ions not being fragmented. The advantages of the DIA-WideSIM/MS^2^ approach is the completeness of the analysis and the archival nature providing for re-analysis of data to probe for newly found or expected adducts in previously analyzed samples. A negative of the DIA analysis is that fragmentation data is limited to MS/MS and principally only the adduct-identifying fragment ion is considered. There is the potential for generation of a pseudo-MS^2^ fragmentation spectrum either by peak picking in the MS/MS spectra, and co-alignment with the identified chromatographic peak of the adduct in the full scan data, or manual interrogation of the data. In addition, while DIA has the potential to provide more thorough coverage than the DDA approach, it requires advanced data analysis [47,48,49,50], which is currently not available for DNA adductomics analysis, as well as mastery of the data acquisition parameters necessary for development of the data acquisition methodologies.

## 4. Adductomic Studies

The field of DNA adductomics has been recently reviewed and [29,51,52] Table 2 summarizes the LC-MS based DNA adductomics analyses performed to date. Three recent studies [53,54,55] have used the conventional low resolution, nominal mass MS^2^ approach for DNA adductomics, but the trend since our previous review is to use HRAM data acquisition for DNA adductomic experiments. Two studies [33,34] have relied on full scan HRAM data acquisition and a self-generated DNA adduct database searching for DNA adductomic analysis, whereas another recent study [35] used HRAM DIA data acquisition using the simultaneous acquisition of fragment ions resulting from high and low collision energy (MS^E^). Our approach [36,37,38] takes advantage of DDA HRAM data acquisition with MS/MS/MS fragmentation upon observation of neutral loss of dR (Figure 2A) or base (Figure 2B). The new studies using HRAM detection are discussed briefly in Section 5.

### Need for Methodology Comparisons

Comparisons of the various methodologies would be very useful for deciding upon an optimal DNA adductomic approach for a given experiment. It seems unlikely that one single approach would be best in all contexts, and considerations such as differences in analytical goals (e.g., targeted or untargeted), DNA amounts available, DNA adduct levels, hydrophobic vs. hydrophilic adducts, instrument availability, etc. will need to considered to determine the best approach to use. Comparisons of previously published studies are difficult if not impossible. For example, comparing analyses performed by DDA-CNL/MS^3^ analysis using low resolution/nominal mass detection with an ion trap instrument [56] and high resolution/accurate mass detection with an Orbitrap instrument [36] would be informative, especially in the context of our advocacy for HRAM data acquisition for DNA adductomics, but these two studies used different sources of DNA to perform their proof-of-principle investigations. Ideally, direct comparisons of various DNA adductomic methodologies using identical samples would provide a true measure of their relative analytical power. Turesky and coworkers have recently performed a comparison of targeted DNA adductomics methodologies of Orbitrap-based DIA-WideSIM/MS^2^ and DDA-CNL/MS^3^ analyses and triple quadrupole-based CNL and pseudo-CNL analyses, all four of which were performed on the same samples with identical chromatography and ion source conditions [57]. Levels of synthetic DNA adduct standards were spiked in calf thymus DNA and analyzed with the different methods. The complete results of this study are beyond the scope of this paper but the performance of the four approaches were DIA-WideSIM/MS^2^ > DDA-CNL/MS^3^ > pseudo-CNL > CNL, where the number of adducts detected were 12, 7, 2, 0, respectively, out of 15 at the lowest level of spiking (4–8 adducts per 10^9^ nucleotides).

## 5. New HRAM DNA Adductomic Studies

### 5.1. Untargeted and Targeted Nanospray HRAM CNL-MS^3^ Analysis

The DNA adductomic methodology using HRAM CNL-MS^3^ detection, a relatively new approach for broad-based screening of DNA adducts, has emerged [36] and efforts are underway to improve the basic methodology, tailor the approach to specific applications, and demonstrate its capabilities [37,38]. The basic method involves HRAM full scan detection followed by DDA MS^2^ fragmentation and subsequent MS^3^ fragmentation of MS^2^ events for which the neutral loss of the deoxyribose moiety was observed. The presence of the MS^3^ fragmentation event serves as an indicator of probable adduct detection. The initial proof-of-principle analysis, expanding upon the work of Turesky and coworkers with ion trap detection [56], was performed with incorporation of HRAM detection (5 ppm) and nanospray ionization (300 nL/min) to further empower the CNL-MS^3^ approach [36]. The method was optimized using a mix of 18 synthetic DNA adduct standards which included adducts of all 4 bases. Liver tissue from mice exposed to nitrosamine 4-(methylnitrosamino)-1-(3-pyridyl)-1-butanone (NNK) was analyzed with detection of both previously characterized and putative DNA adducts. The methodology was refined [37] for screening of anticipated and unknown adducts induced in cells treated with a chemotherapeutic DNA alkylating agent (PR104A, an experimental nitrogen mustard prodrug under investigation for treatment of leukemia) by incorporating neutral loss triggering of the four DNA bases (Figure 2B) into the methodology. In addition, an extensive ion mass list including all suspected ions from the alkylating agent and metabolites along with all four bases, including cross-link adducts, was utilized for data dependent triggering leading to the detection of many mono- and cross-linked adducts which had not been observed previously. Most recently, the method was used to successfully identify and semi-quantify endogenous and exogenous DNA adducts in the lung of mice exposed to NNK and the proinflammatory agent LPS to observe an adductomic profile [38]. This methodology utilized an extensive list of parent ions from previously observed endogenous adducts as well as suspected adducts resulting from exposure to NNK, and took advantage of an advanced hybrid Orbitrap instrumentation (Fusion).

### 5.2. Untargeted HRAM MS^E^ Analysis

Totsuka and coworkers developed a comprehensive DNA adductomic analysis for DNA samples derived from the lungs of mice exposed to nanosized-magnetite (MGT) using an MS^E^ approach [35] to identify DNA adducts resulting from inflammation. Briefly, the MS^E^ approach is data independent acquisition (DIA) scanning mode where all ions of interest undergo low and high energy fragmentation and the subsequent ion signal undergoes data analysis to reconstitute fragmentation spectra for individual ions with subsequent identification of the corresponding analytes. Data was acquired with a Waters Xevo QTOF mass spectrometer with a mass range of *m*/*z* 50–1000 and a scan duration of 0.5 s (1.0 total duty cycle). The resolution is not reported, however the data analysis was performed with a mass tolerance of 0.05 Da. Reversed phase UPLC separation was performed using a 1.0 mm ID × 150 mm C18 column with 1.7 μm particles and a flow rate of 25 μL/min. In total they detected 30 and 42 types of DNA adducts in the vehicle control and MGT-treated groups, respectively. They performed principal component analysis (PCA) against a subset of DNA adducts and several adducts, which are deduced to be formed by inflammation or oxidative stress (e.g., etheno-deoxycytidine (εdC)), revealed higher contributions to covalent DNA modification resulting from MGT exposure. The levels of εdC were quantified by LC-MS/MS and found to be significantly higher in MGT-treated mice than those of the vehicle control. This analysis is the first example of DIA data acquisition for DNA adductomics analysis.

### 5.3. Targeted HRAM Full Scan Analysis

An alternative approach to relying upon MS/MS and MS/MS/MS data as confirmation of adduct identity is to develop a DNA adduct database in a targeted DNA adductomics approach and rely upon the accurate mass of the adduct parent ion (MS^1^) as an indication of adduct identity. This is the approach developed [33,34] by Vanhaecke and coworkers, in which they created a database of 123 diet-related DNA adducts. This approach used acid-hydrolysis of DNA such that the deoxyribose moiety, which is commonly used in DNA adductomic analysis as an indicator of DNA adduct identity, is not present. The exact mass (10 ppm) and ^12^C/^13^C ratio of the parent ion was used as confirmation of putatively identified adducts identity with full scan data collected using an Orbitrap detector at a resolution of 100,000 and 3 microscans per spectrum. The methodology was used [33] to analyze in vitro beef digests using fecal microbiota from human subjects and found various DNA adduct profiles consisting of adducts formed from DNA alkylation, oxidation, and reaction of DNA nucleobases by lipid peroxidation products.

### 5.4. Adduct-Tagging MALDI Ionization Approach

A DNA adductomic approach differing significantly from the conventional methodology described above has been performed, whereby nucleotides are derivatized with benzoylhistamine. Analysis is performed using MALDI-TOF and MALDI-TOF/TOF detection with adduct identification based upon phosphate-specificity of the tagging, detection of adducts as a pair of ions, and measurement of fragment ions characteristic of the presence of deoxyribose or ribose [70,71].

## 6. Challenges

In vitro DNA adductomics analysis can provide useful information regarding a given biological system, however we feel the ultimate goal should be to analyze in vivo systems and ultimately human samples. There are several challenges to making in vivo DNA adductomics analysis a robust and powerful approach to screening for DNA modification, and they are discussed below.

### 6.1. Selectivity

We feel that the path forward to fully realizing the promise of DNA adductomics, namely the comprehensive assessment of DNA modification in a variety of exposure contexts at trace levels in biological matrices, is to utilize the analytical power of HRAM and MS^n^ data acquisition available with the ever-improving modern LC-MS instrumentation. Targeted DNA adduct analysis, while typically performed with triple quadrupole instrumentation (low resolution nominal mass detection), relies upon stable isotopically labeled internal standards, not only for quantitation but just as importantly for confirmation of identity of the analyte being measured. In our experience, there are frequently peaks in these MS/MS chromatograms which are either adjacent or co-eluting with the analyte of interest, especially at the lower levels found in in vivo samples, and could and probably would be attributed to the analyte to be measured if not for the internal standard. While the triple quadrupole MS^2^ approaches or MS^1^ HRAM strategies may be useful for in vitro applications, where exposures are well defined and usually at higher levels, we feel that for in vivo applications, the power of HRAM MS^n^ is needed to provide the certainty required for analysis of DNA adducts in the absence of internal standards.

### 6.2. Sensitivity

One of the main factors limiting the sensitivity for screening DNA modifications is the amount of DNA available for analysis, which is especially true in human blood or biopsy samples, but can also be case for tumor and tumor adjacent tissue where the samples are precious and only a small amount acquired may be made available for DNA adductomic analysis.

The sample cleanliness and chemical complexity of the samples affects not only the selectivity but also the sensitivity. For traditional MS^2^ based analysis, the background signal is directly related to the chemical complexity and limits the ability to detect low level adducts. This is less of an issue for HRAM analyses because of the discrimination provided by accurate mass measurements. Chemical noise can limit the sensitivity of trap-based instrumentation due to the finite capacity of the trap, which limits the ability to see trace level ions in the presence of abundant background ions. In addition, for methods using DDA for untargeted detection, the chemical complexity of the sample limits the ability to detect unknown trace level adducts since the scanning speed of the instrumentation is insufficient to sample all ions present as the chromatogram. The presence of matrix material can also impact sensitivity by suppression of the ion signal, a chronic problem with electrospray ionization of LC-MS analysis. Therefore, sample preparation needs to be thoroughly optimized and sensitivity maximized through the use of nanospray ionization.

Finally, nanospray ionization is a powerful option for increasing the inherent sensitivity of ESI-LC-MS analysis. The field of proteomics uses this approach nearly universally and this has, over time, made this a routine mode of operation. It is now possible with minimal experience or training to easily operate in nanospray mode. We feel nanospray should be the default mode of operation for DNA adductomics, due to the trace levels of adducts and the often limited amount of DNA available for analysis.

### 6.3. Quantitation

Accurate absolute quantitation by LC-MS requires the use of stable isotope-labeled internal standards of the analytes of interest. This is either not possible in the case of untargeted DNA adductomics or impractical in the case of targeted DNA adductomics monitoring for hundreds of adducts at a time. Fortunately, typically absolute quantitation is not necessary to draw the conclusions needed to answer the scientific questions that DNA adductomics is designed to answer. Namely, which DNA adducts are formed at measureable levels and what are the relative amounts of the individual adducts across the samples analyzed. The use of internal standards accounts for several issues, including ionization efficiency variation across analytes, possible losses during sample preparation (recovery), and ion suppression/enhancement due to sample matrix components [15,16]. Relative quantitation of individual analytes across samples is possible if either there is no ion suppression/enhancement and 100% recovery or the ion suppression/enhancement and recovery are consistent across samples. The probability of this being the case is most likely related to the complexity of the matrix. In the case of DNA adduct analysis, the matrix is much simpler than other common biological matrices commonly analyzed by LC-MS such as urine or plasma. The complexity of the DNA samples should consist of the unmodified bases, which are very hydrophilic and therefore elute much earlier than many adducts, hydrolysis enzymes, and any impurities in the enzymes and unpolymerized constituents entering the sample solution when using plastic components, such as solid-phase extraction cartridges and molecular weight filters for sample preparation [29,36,72]. The impurities due to the use of hydrolysis enzymes can be nearly eliminated, or at least greatly reduced, by careful enzyme source and vendor selection, cleaning of the enzymes prior to use, and determining the minimal enzyme necessary for the analysis [72,73]. Impurities due to the use of plastics can be greatly reduced or eliminated by avoiding the use plastics either entirely or as much is practically possible and by careful type/vendor selection of consumables used for the experiments.

A relative quantitation strategy, which will account for variable ion suppression/enhancement and recovery, has recently been demonstrated with the DNA adductomic analysis of a cell-based system [37,74]. It involves the generation of a mix of DNA adducts by the treatment of cells with an isotopically labeled version of the genotoxic substance of interest. This mix of isotopically labeled adducts was used for relative quantitation of many adducts in subsequent experiments by spiking the labeled adduct internal standard mixture into cell, animal, or human samples.

Additionally, relative quantitation is also possible, using a peak-area-based labeled free quantitation method. This approach is based on integrating the adduct precursor ion chromatogram peak areas in the full scan and on normalizing the signal intensity to the amount of DNA used for the analysis and to a quantitative reference, a labeled internal standard added in constant amounts to each sample.

Lastly, if it is determined that absolute quantitation or more precise relative quantitation is necessary, the targeted quantitation of those adducts of interest can be performed via the traditional quantitation approach after synthesis of the labeled internal standards.

### 6.4. Ease of Data Analysis

Software development for advanced HRAM data analysis has strived to keep pace with advances in mass spectrometry instrumentation to take full advantage of the technology. Unfortunately, the advances have focused upon proteomics, metabolomics, metabolite analysis, lipidomics, etc. and have not been geared toward the type of analysis required for DNA adductomics. Therefore, further development of software tools for the data analysis is necessary to interpret the results coming from DNA adductomics experiments. Software solutions are needed for both DDA and DIA data. In the case of DNA adductomics DDA data, software tools for the recognition, tabulation, and display of fragmentation data which corresponds to the neutral loss of deoxyribose and bases (see Figure 2A,B) would help with the automated and high throughput analysis of the data. For example, ideally for our DDA-CNL/MS^3^ methodology [36,37,38], for each putative adduct identification, an output displaying the MS/MS and MS/MS/MS spectra as well as the integrated extracted ion chromatogram would be very useful, and tabulation of this data in a searchable format would be ideal. In the case of the DIA-Wide SIM/MS^2^ methodology we recommend, software tools are needed which can perform a variety of tasks in an automated fashion, such as perform peak picking, extract ion chromatograms for the SIM and MS/MS data, integrate the resulting peak areas, compare retention times and mass differences, and tabulate and display the results.

## 7. Summary

The identification and structural characterization of DNA adducts in human tissues can be used to either identify specific genotoxic exposures or confirm that suspected exposures have occurred and led to DNA modification. Quantitation of these DNA adducts can be used to assess the extent of this damage. Recent advances in the field of mass spectrometry have led to the development of DNA adductomic methods which can be used to comprehensively identify, characterize, and semi-quantify the DNA adducts produced in an in vivo system or in human samples. We feel that the most advanced, sophisticated aspects of this new era of mass spectrometry should be harnessed to make this type of analysis a powerful tool for screening for DNA modification characterization in biologically relevant contexts. In addition, careful use of negative controls and scrutiny of HRAM data is necessary to assure signal is rightfully attributed to putative DNA adducts. As in the other “-omics” methodologies, we envision different basic approaches being used depending upon the needs of the specific experiments. For example, HRAM DIA-Wide SIM/MS^2^ analysis might be better suited for screening for large numbers of known adducts, whereas HRAM DDA-CNL/MS^3^ analysis may be better suited for identifying unknown DNA adducts.

The ultimate goal of DNA adductomics [29] is to characterize the modifications of DNA as a profile of specific adducts, rather than focusing only on a few adducts at a time. There are many applications of DNA adductomic analysis, including investigating the genotoxic effect of exposures from the environment [33,34,35,36,41,51,53,54,55,56,59,60,61,62,63,65,66], as well as endogenous adduct formation [38,64,67]. It can be used to investigate mechanisms of actions of genotoxic chemotherapeutic drugs [37,68,69], and the mutagenicity potential of pharmaceuticals or supplements in the context of cancer risk. It can be used in drug design for development of DNA alkylating chemotherapeutic agents, both in terms of maximizing the genotoxicity to cancer cells as well as minimizing the genotoxicity to healthy cells [37]. The ability to broadly screen for DNA adducts can also be used for a “precision medicine” approach to chemotherapeutic drug treatment [74]. Screening for DNA modification by non-cancer therapeutic drugs, as well as minimizing toxicity during drug development, are also possible applications. Lastly, screening of epigenetic changes [16] is also a possible use for DNA adductomics, whereby all known epigenetic modifications to DNA bases could be monitored while simultaneously screening for unknown modifications.

Currently, DNA adductomics offers the potential to fully characterize the chemical modification of DNA by detection and relative quantitation of known and unknown DNA adducts, providing information regarding the exposures which have occurred, resulting genotoxic effects, and, more importantly, elucidating mechanisms of interaction between chemicals and DNA. Overall this approach provides crucial complementary information to that acquired from mutagenicity assays.

The critical DNA modifications resulting from exposure to a particular compound may be on specific DNA sequences or chromatin structures. DNA adductomics requires the hydrolysis of DNA to allow for the analysis of the modified nucleosides, and therefore any information regarding the sites of the modifications is lost. For now, DNA adductomics should be combined with genomic-wide sequencing to correlate DNA adduct formation with biologically important mutations. However, the promising trend of improving instrumentation and molecular biology techniques leads us to believe that in the future we will be able to perform this analysis on specific sequences and targeting specific genes.

## Figures and Tables

**Table 1:** Summary of MS^2−3^ data dependent acquisition (DDA) and MS^2^ data independent acquisition (DIA) scanning modes.

Approach	Method	Scan Events	Frequency	Adduct Detection
DDA CNL/MS^3^	Targeted	Full Scan	Continuous	MS/MS/MS Triggered Event
		MS/MS	Ions included in a list	
		MS/MS/MS	MS/MS ions selected by loss of 116.0474	
	Untargeted	Full Scan	Continuous	MS/MS/MS Triggered Event
		MS/MS	Most abundant ions	
		MS/MS/MS	MS/MS ions selected by loss of 116.0473	
DIA Wide SIM/MS^2^	Targeted	Full Scan	Continuous	Post-run data analysis on ions from a list (characterized by co-eluters with NL = 116.0473)
		MS/MS	Continuous	
	Untargeted	Full Scan	Continuous	Post-run data analysis (any co-eluters with NL = 116.0473)
		MS/MS	Continuous	

**Table 2:** Summary of published LC-MS-based DNA adductomics studies.

Approach	Instrument	Sample Type	Adduct Type/Origin	Strengths	Weaknesses	Details	Reference
CNL	DF-EB/Q	Reaction with nucleosides	PGE ^c^ (industrial chemical)	High resolution, First example of DNA adductomic analysis	Simplistic model	Nucleoside reacted with chemical of interest	Claereboudt et al., 1990 [32]
	Triple Quad	Synthetic standards	Arylamine (industrial chemical)	Early report of DNA adductomics	Nominal mass measurement and lack of fragmentation data	Analysis of synthetic standards only	Bryant et al., 1992 [58]
		In vitro reaction	PhIP ^a^ (food)	Comparison made with ^32^P-postlabeling		-	Vouros et al., 1995 [59]
		In vitro reaction and Animal tissues	IQ ^b^ (food)	First example of nanospray ionization		-	Vouros et al., 1999 [41]
		Irradiated cells (human monocyte)	Radiation-induced	Only example of analysis of adducts due to exposure to radiation		-	Ravanat et al., 2004 [60]
		In vitro reaction	PAH (environmental/industrial exposure)	Automated data analysis		Small mass range (500–650 Da)	Singh et al., 2010 [61]
		Reaction with oligonucleotide	PGE ^c^, SO ^d^ (industrial chemicals)	-		Limited to oligonucleotides	Feng et al., 2016 [53]
		Treated cells (from ovarian follicles)	PAH ^e^ (environmental/industrial exposure)	-		-	Feng et al., 2016 [54]
Pseudo-CNL		Human lung tissue	Screening for all DNA modifications	Adductome map data analysis		-	Matsuda et al., 2006 [62]
		Human lung and esophagus tissue	Screening for all DNA modifications	Seven adducts unambiguously detected		-	Matsuda et al., 2007 [63]
		Various human tissues	LPO-induced (endogenous)	Reported lipid peroxidation-derived adducts in humans		-	Matsuda et al., 2010 [64]
		Quorn, button mushrooms, brewer’s yeast	Food	-		Only 7 SRM transitions per injection	Berdal et al., 2010 [65]
		Treated cells (Chinese hamster)	Micronucleus test-positive compounds	First comparison to micronucleus test		-	Yagi et al., 2011 [66]
		Human gastric mucosa	LPO (endogenous)	-		-	Matsuda et al., 2013 [67]
		Soil Bacterium	Screening for all DNA modifications	First DNA adductomic study of bacterial DNA		-	Kanaly et al., 2015 [55]
DD-MS^2^	Q-TOF	Treated cells (immortalized human T lymphocyte)	Melphalan (chemotherapy drug)	First example of MS^2^ spectral data acquisition	No MS^3^ fragmentation data, accurate mass data not reported	-	Esmans et al., 2004 [68]
MS^E^ (HRAM)		Mouse lung tissue	Magnetic nanoparticles	First application of MS^E^	No MS^2^ or MS^3^ data, reported accurate mass data limited to 10 mmu	-	Totsuka et al., 2015 [35]
Full Scan (HRAM)	Orbitrap	Human colon tumor tissue	Diet-related	-	-	Diet-related DNA adduct database, acid hydrolysis resulting in nucleobase adducts	Vanhaecke et al., 2015 [34]
		In vitro microbiota meat digests	Diet-related	-	-	Utilized methodology developed in [34]	Vanhaecke et al., 21016 [33]
DD-CNL-MS^3^	Ion Trap	Treated cells (human hepatocytes) Rat liver Human buccal cells	4-ABP ^f^, MeIQx ^g^ Tobacco constituents	Human samples examined, First example of MS^3^ data acquisition	No accurate mass measurements	-	Turesky et al., 2009 [56]
		Treated cells (human colon adenocarcinoma)	Illudin S (chemotherapeutic natural product)	-		Used similar method to Turesky [56]	Sturla et al., 2013 [69]
DD-CNL-MS^3^ (HRAM)	Orbitrap	Mouse liver tissue	Tobacco constituents	Combination of HRAM, MS^3^ and nanospray	Extensive sample purification and multiple injections	-	Balbo et al., 2014 [36]
		Treated cells (human colon adenocarcinoma)	DNA alkylating drug	First targeted approach	-	-	Balbo et al., 2015 [37]
		Mouse lung tissue	Endogenous adducts	HRAM MS^3^ data acquisition	-	-	Balbo et al., 2017 [38]

^a^ 2-Amino-1-methyl-6-phenylimidazo[4,5-b]pyridine (PhIP); ^b^ 2-Amino-3-methylimidazo[4,5-f]quinolone (IQ); ^c^ Phenyl glycidyl ether (PGE); ^d^ Styrene-7,8-oxide (SO); ^e^ Polycyclic aromatic hydrocarbons (PAH); ^f^ 4-Aminobiphenyl (4-ABP); ^g^ 2-Amino-3,8-dimethylimidazo[4,5-f]quinoxaline (MeIQx).

## Abbreviations

LC-MS	Liquid chromatography—mass spectrometry
LC-MS^2^	Liquid chromatography—tandem mass spectrometry
LC-MS^n^	Liquid chromatography—multistage fragmentation mass spectrometry
HRAM	High resolution/accurate mass
MALDI	Matrix assisted laser desorption ionization
TOF	Time-of-flight
UPLC	Ultra high pressure liquid chromatography
Q-TOF	Quadrupole-Time-of-flight
DDA	Data dependent acquisition
DIA	Data independent acquisition
CNL/MS^3^	Constant neutral loss/triple stage mass spectrometry
SIM/MS^2^	Selected ion monitoring/tandem mass spectrometry
NNK	Nitrosamine 4-(methylnitrosamino)-1-(3-pyridyl)-1-butanone
LPS	Lipopolysaccharide
PCA	Principal component analysis
MALDI-TOF	Matrix assisted laser desorption ionization—time-of-flight
MALDI-TOF/TOF	Matrix assisted laser desorption ionization—time-of-flight/time-of-flight
